# Estimated Annual Spending on Aducanumab in the US Medicare Program

**DOI:** 10.1001/jamahealthforum.2021.4495

**Published:** 2022-01-14

**Authors:** John N. Mafi, Mei Leng, Julia Cave Arbanas, Chi-Hong Tseng, Cheryl L. Damberg, Catherine Sarkisian, Bruce E. Landon

**Affiliations:** 1 Division of General Internal Medicine and Health Services Research, David Geffen School of Medicine at UCLA, Los Angeles, California; 2 RAND Corporation, Santa Monica, California; 3 VA Greater Los Angeles Healthcare System, Los Angeles, California; 4 Division of General Medicine and Primary Care, Beth Israel Deaconess Medical Center, Harvard Medical School, Boston, Massachusetts; 5 Department of Health Care Policy, Harvard Medical School, Boston, Massachusetts

## Abstract

This cross-sectional study examines upper bound and lower bound annualized Medicare costs for administering aducanumab to beneficiaries with the approved indications of mild cognitive impairment or mild dementia.

## Introduction

The US Food and Drug Administration’s June 2021 decision to approve aducanumab for treatment for Alzheimer dementia raised concerns that a drug with uncertain benefit and high cost could, in aggregate, threaten Medicare's solvency. In response to these concerns, Biogen recently announced a 50% annual drug price reduction from $56 000 to $28 200 per patient. Preliminary US spending estimates either used extrapolated Alzheimer dementia prevalence data from 2012 or did not explicitly quantify ancillary costs, such as additional diagnostic imaging to monitor the amyloid-associated imaging abnormalities (ARIAs) that occur in 41% of treated patients, and did not incorporate the recently announced price reduction.^1,2,3^ We estimated upper bound and lower bound annualized Medicare costs for administering aducanumab to beneficiaries with the approved indications of mild cognitive impairment (MCI) or mild dementia, focusing on the degree to which associated ancillary health services affect spending.^1^

## Methods

For this cross-sectional study, we used validated cognitive measures from the 2016 Health and Retirement Study, a nationally representative survey of older adults, to identify Medicare Part B beneficiaries 65 years or older with MCI or mild dementia with either Medicare fee-for-service or Medicare Advantage coverage (Figure^4,5^). Institutional review board approval was provided by the University of California, Los Angeles, and informed consent was waived because of the study's design as a secondary analysis of publicly available, deidentified data. We followed the Strengthening the Reporting of Observational Studies in Epidemiology (STROBE) reporting guidelines for reporting cross-sectional studies.^4,5^

**Figure.**
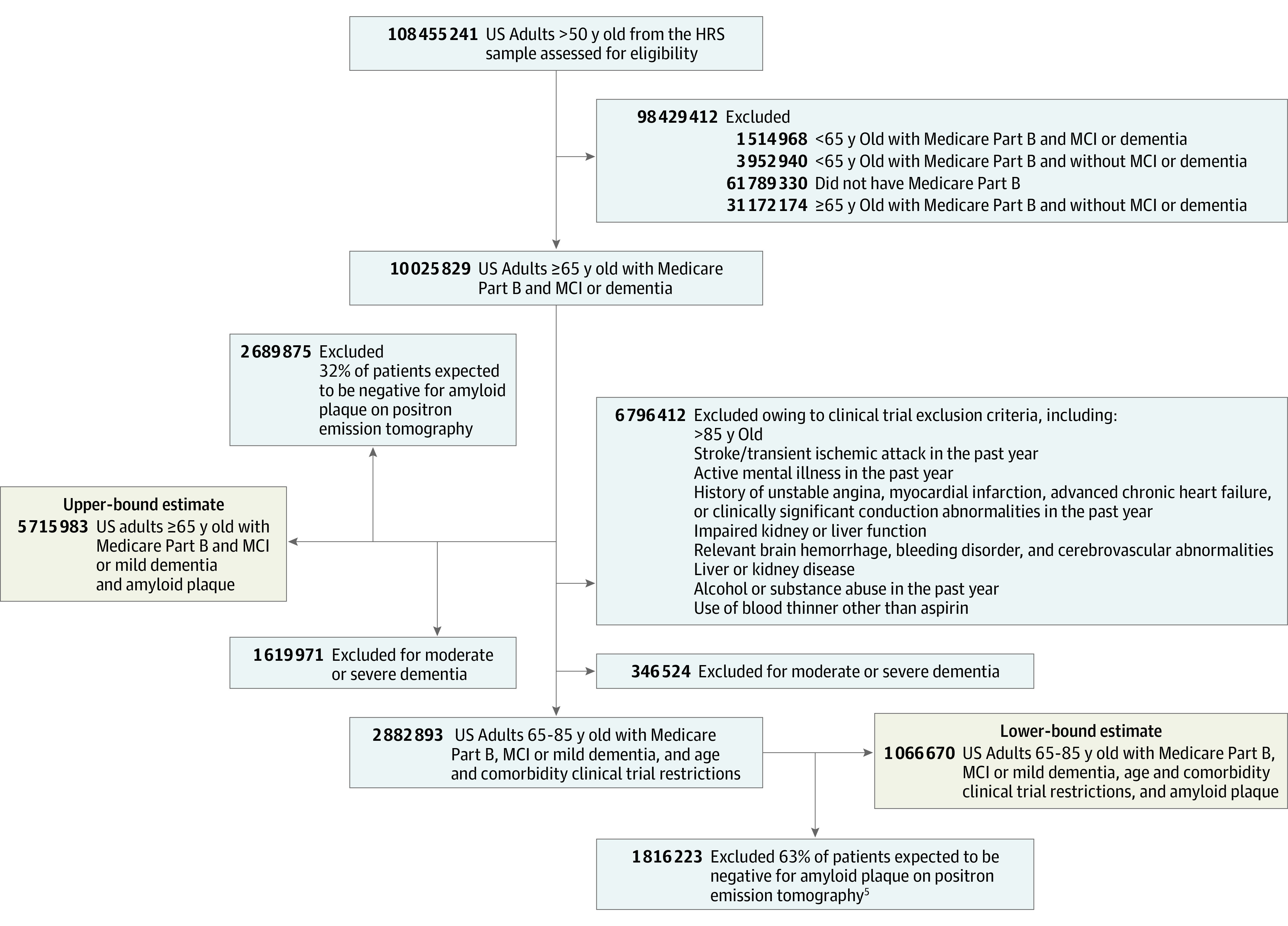
Diagram of Estimated Range of Older US Adults With Mild Cognitive Impairment (MCI) and Dementia Because of Alzheimer Dementia in the 2016 Health and Retirement Study (HRS) Core Sample We used data from the 2016 HRS, a nationally representative survey of adults older than 50 years that studies the health and economic changes of aging. The national sample was obtained biennially at the household level and used a multistage, national area-clustered probability sampling frame. We identified 8396 HRS participants who represented approximately 41.2 million (95% CI, 39.2-43.1 million) US adults 65 years or older with Medicare Part B coverage in 2016, including Medicare fee-for-service and Medicare Advantage beneficiaries (beneficiaries must enroll in Medicare Part A and Part B to join a Medicare Advantage plan). To identify eligible patients with MCI or dementia, we used a 27-point cognitive scaling score (including immediate and delayed 10-noun free recall testing, serial 7 subtraction testing, and a backward count from 20).^4^ This scale classified participants with scores ranging from 0 to 6 as having dementia and those with scores ranging from 7 to 11 as having MCI. For participants represented by a proxy, we used an 11-point scale that classified participants with scores ranging from 6 to 11 as having dementia and those with scores ranging from 3 to 5 as having MCI. Based on prior work, we also further subclassified dementia severity using the following threshold scores for the 27-point and 11-point scales, respectively: (1) mild dementia, 5 to 6 and 6; (2) moderate dementia, 3 to 4 and 7; and (3) severe dementia, 0 to 2 and 8 to 11. While these and similar measures have previously demonstrated strong validity,^4,5^ we performed an additional sensitivity analysis showing that these dementia stages showed a strong association with the presence of an informal caregiver, which is a proxy for functional status (eTable 1 in the Supplement).

The lower bound estimates of patients eligible for treatment with aducanumab assume prescribers would apply clinical trial inclusion criteria to adults with MCI or mild dementia with amyloid plaque on positron emission tomography imaging results. The upper bound estimate includes patients with MCI or mild dementia and plaque without age or comorbidity restrictions to reflect potential off-label prescribing.^1^ Between 37% to 68% of patients with MCI or dementia have plaque according to population studies (we used 37% for the lower bound and 68% for the upper bound estimate).^6^

We quantified drug costs from patient weights and ancillary costs, such as additional magnetic resonance imaging scans, using amyloid-associated imaging abnormalities rates from clinical trials and US Food and Drug Administration recommendations (Table).^1,2^ We multiplied annualized per-person costs by lower bound and upper bound population estimates of MCI or mild dementia prevalence. Medicare would pay 80%, and the remaining 20% coinsurance would be paid by beneficiaries, private supplemental plans, and/or state Medicaid programs. While cost sharing may vary for Medicare Advantage beneficiaries, the total costs will remain the same regardless of the cost-sharing rules. The eAppendix and eTables 1 to 3 in the Supplement provide further details on dementia identification, weight-based drug cost estimates, and cost analysis assumptions.

**Table.** Estimated Annual Spending on Treatment With Aducanumab Among Older US Adults With MCI or Mild Dementia Because of Alzheimer Disease in the 2016 HRS Core Sample^a^

Characteristic	No. of events per patient-year	$					
		Estimated per-person unit costs	Annualized Medicare cost per patient (80% of cost)	Annualized coinsurance cost to beneficiaries, private supplemental plans and/or state Medicaid plans (20% of cost)	Anticipated annualized per-person out-of-pocket cost ranges	Annual Medicare cost estimate (millions)	
						Lower bound (n = 1 066 670 [95% CI, 0.95-1.2 million])	Upper bound (n = 5 715 983 [95% CI, 5.3-6.2 million])
PET scan	1.5	1535.00	1842.00	460.50	0-460.50	1964.8	10 528.8
Aducanumab	12	2313.28	22 207.49	5551.87	0-5551.87	23 688.1	126 937.6
Intravenous infusion	12	198.12	1901.95	475.49	0-475.49	2028.8	10 871.5
Neurology/geriatrics visit	4	141.04	451.33	112.83	0-112.83	481.4	2579.8
Routine MRI scan of brain	2	277.56	444.10	111.02	0-111.02	473.7	2538.4
Apo E serum testing	1	99.00	79.20	19.80	0-19.80	84.5	452.7
Moderate to severe ARIA							
Additional MRI scan of brain	0.78	277.56	173.20	43.30	0-43.30	184.7	990.0
Additional neurology visit	0.78	141.04	88.01	22.00	0-22.00	93.9	503.1
Symptomatic mild ARIA							
Additional MRI scan of brain	0.108	277.56	23.98	6.00	0-6.00	25.6	137.1
Additional neurology visit	0.108	141.04	12.19	3.05	0-3.05	13.0	69.7
Hospitalization for severe AE	0.02	14 700.00	235.20	58.80	0-58.80	250.9	1344.4
Subtotal costs	NA	NA	27 458.64	6864.66	0-6864.66	NA	NA
Total costs accounting for 9.1% population attrition over time (95% CI)	NA	NA	NA	NA	NA	27 896.3 (24 745.1-31 047.6)	149 488.6 (137 690.7-161 286.5)

Abbreviations: AE, adverse event; Apo E, apolipoprotein E; ARIA, amyloid-related imaging abnormality; HRS, Health and Retirement Study; MCI, mild cognitive impairment; MRI, magnetic resonance imaging; NA, not applicable; PET, positron emission tomography.

^a^ We used data from the 2016 HRS, a nationally representative survey of adults older than 50 years. We identified 8396 participants who represented approximately 41.2 million (95% CI, 39.2-43.1 million) US adults 65 years or older with Medicare Part B coverage in 2016, including Medicare fee-for-service and Medicare Advantage beneficiaries (beneficiaries must enroll in Medicare Part A and Part B to join a Medicare Advantage plan). Using validated measures of cognition, we identified approximately 1.1 million beneficiaries with MCI or mild dementia and plaque with age or comorbidity restrictions (lower bound estimate) and 5.7 million beneficiaries with MCI or mild dementia and plaque without age/comorbidity restrictions (upper bound estimate). We derived drug costs using HRS patient weights assuming the full 10 mg/kg per month administration at a cost of $846.00 for a 300 mg/3 mL vial and $479.40 for a 170 mg/1.7 mL vial, using the most cost-efficient dosing approach. Incorporating patient weights yielded an estimated annual drug cost of $27 759.36. We then quantified ancillary costs by multiplying the 2021 Medicare physician fee schedule reimbursement rates (adjusted for facility fees) for each anticipated associated health service per year by the lower bound and upper bound estimates of the eligible populations (and the 95% CIs of each estimate) and accounting for the 9.1% overall attrition rate from the trial data. Anticipated associated health services were obtained by pooling data from 2 phase 3 clinical trials from the US Food and Drug Administration documentation,^3^ which showed among treated patients a moderate to severe ARIA rate of 26% and a 3.6% symptomatic mild ARIA rate (with an average duration of 12 weeks, each ARIA would require 3 additional MRIs), with severe AEs requiring hospitalization in 2% of patients. According to Medicare Part B fee-for-service rules, Medicare pays 80% of the physician fee schedule rate, while the remaining 20% is paid either fully or partly by the beneficiaries, private supplemental plans, or state Medicaid programs. For Medicare Advantage beneficiaries, this coverage split varies, and we were not able to provide a more precise estimate. Therefore, this analysis assumes an 80%/20% coverage split for all patients, albeit total costs remain the same regardless of the coverage split rules. Specifically, the Medicare beneficiaries in this sample without supplemental coverage were obligated to pay 20% coinsurance for Part B services, or $6864.66. Per Medicare coverage policy, physicians cannot bill dual-eligible Medicare-Medicaid beneficiaries who are qualified Medicare beneficiaries for Part A or B cost sharing, including deductibles, copays, and coinsurance, making the lower range of cost sharing $0 for this type of dual-eligible beneficiary. We cannot provide more precise estimates of out-of-pocket costs, particularly for Medicare Advantage and supplemental private insurance, until private coverage policies are announced. Finally, when we calculated the proportion of ancillary costs at the individual level, this slightly reduced the amount to 19.1% of total estimated costs. This individual-level estimate of 19.1% is slightly lower than the population-level estimate of 19.4% because of patient attrition. See the eAppendix in the Supplement for further details.

We analyzed data using SAS, version 9.4 (SAS Institute). We accounted for survey clustering and adjusted results by survey weights for the national representativeness and response rate.

## Results

We identified 8396 participants representing approximately 41.2 million (95% CI, 39.2-43.1 million) US adults 65 years or older with Medicare Part B coverage in 2016. Total annualized per-person weight-based drug costs equaled $27 759.36; ancillary costs equaled $6563.94. For the lower bound estimate, if 25% of the 1 066 670 (95% CI, 0.95-1.2 million) eligible patients with MCI or mild dementia and plaque with clinical trial age and comorbidity restrictions received treatment with aducanumab, Medicare would pay $7.0 billion (95% CI, $6.2-$7.8 billion) each year. For the upper bound estimate, if 25% of the expanded population of 5 715 983 (95% CI, 5.3-6.2 million) eligible patients with MCI or mild dementia and plaque received treatment with aducanumab, Medicare would pay $37.4 billion (95% CI, $34.4-$40.3 billion). Ancillary health services comprised 19.4% of total population spending estimates.

## Discussion

In this nationally representative analysis, we identified between 1.1 to 5.7 million Medicare beneficiaries who are potentially eligible to receive treatment with aducanumab. Ancillary health services comprised nearly 20% of total population spending estimates, suggesting that prior analyses underestimated the anticipated costs of aducanumab.^3^

This study has limitations. We used plaque rates from population studies rather than actual scans on Health and Retirement Study participants.^6^ Our validated approach of identifying dementia prevalence may have misclassified some cases.^4^ Surveys may be less reliable among participants with dementia, although participants’ proxies were frequently available.^4^ Furthermore, we did not account for potential future changes to drug acquisition costs or for societal costs, such as caregiver burden or health system capacity limitations.

## Conclusions

The findings of this cross-sectional study suggest that the drug and ancillary costs of aducanumab could seriously strain Medicare’s budget, raising questions of how to maximize the value of public dollars, particularly for a drug with unknown benefit and known risk of patient harm.

## Notes

Supplement. eAppendix. eTable 1.Presence of Reported Informal Caregivers Stratified by Dementia Stage eTable 2. Comparison of Measured Weight and Self-Reported Weight Distributions Among HRS Participants in 2016 eTable 3. Weight-Dependent Drug Dosage Calculated Costs eReferences.
