# Errors in Table, Figure, and Body of the Article

**DOI:** 10.1001/jamahealthforum.2022.0053

**Published:** 2022-02-18

In the Research Letter titled “Estimated Annual Spending on Aducanumab in the US Medicare Program,”^1^ the phrasing of sentences in the Results, Discussion, and footnote of the Table was corrected. Additionally, “22 207.49” was changed to “2313.28” and “46 377.39” to “22 207.49” in the row titled “Aducanumab” in the Table, and corrections were made to the third row, right side section of the Figure, which now begins “6 796 412 Excluded owing to clinical trial exclusion criteria.” This article was corrected on February 18, 2022.

## References

[1] Mafi JN, Leng M, Arbanas JC, . Estimate annual spending on aducanumab in the US Medicare program. JAMA Health Forum. 2022;3(1):e214495. doi:10.1001/jamahealthforum.2021.4495 PMC8903103 35977233

