# Supplementary material for: Estimated Annual Spending on Aducanumab in the US Medicare Program
Source: JAMA Health Forum. 2022 Jan 14;3(1):e214495. doi: 10.1001/jamahealthforum.2021.4495 (PMC8903103; doi:10.1001/jamahealthforum.2021.4495)
Supplement: Supplement. — eAppendix. eTable 1.Presence of Reported Informal Caregivers Stratified by Dementia Stage eTable 2. Comparison of Measured Weight and Self-Reported Weight Distributions Among HRS Participants in 2016 eTable 3. Weight-Dependent Drug Dosage Calculated Costs eReferences. [file jamahealthforum-e214495-s001.pdf]

Supplemental Online Content

Mafi JN, Leng M, Arbanas JC, et al. Estimated annual spending on aducanumab in the US Medicare program. *JAMA Health Forum*. 2022;3(1):e214495.
doi:10.1001/jamahealthforum.2021.4495

eAppendix.
eTable 1. Presence of Reported Informal Caregivers Stratified by Dementia Stage
eTable 2. Comparison of Measured Weight and Self-Reported Weight Distributions Among HRS Participants in 2016
eTable 3. Weight-Dependent Drug Dosage Calculated Costs
eReferences.

This supplemental material has been provided by the authors to give readers additional information about their work.

© 2021 Mafi JN et al. *JAMA Health Forum.*

eAppendix

Cohort Design

We followed Strengthening the Reporting of Observational Studies in Epidemiology (STROBE) guidelines for reporting observational cross-sectional studies. To identify eligible patients with mild cognitive impairment (MCI) or dementia, we used a 27-point cognitive scaling score (including immediate and delayed 10-noun free recall testing, serial 7 subtraction testing, and backward count from 20) called the telephone interview for cognitive status (TICS). This scale classified participants with scores ranging from 0-6 as having dementia and those with scores ranging from 7-11 as having MCI. When participants could not respond, their health care proxy answered on their behalf, which helped mitigate the problem of missing data and non-response bias. For participants represented by a proxy, we used an 11-point scale that classified participants with scores ranging from 6-11 as having dementia and those with scores ranging from 3-5 as having MCI. These cognitive measures have previously demonstrated strong validity.[1-3] In our sample, we identified an estimated 10,025,829 Americans age ≥65 years with Medicare Part B coverage and MCI or dementia (see Figure). This analysis includes both traditional Medicare fee-for-service and Medicare Advantage beneficiaries (note, the Centers for Medicare & Medicaid (CMS) require that beneficiaries must enroll in Medicare Part A and Part B in order to join a Medicare Advantage plan[4]). These Medicare Part B beneficiaries had an average age of 79.0 years [SD 0.34], 56.0% female, 67.1% white, 17.7% black, 2.5% other, and 12.8% Hispanic race/ethnicity.

In order to more specifically match the FDA prescription label, which suggests that aducanumab be offered to patients with MCI or mild dementia, we further categorized our dementia cohort as patients with mild, moderate, and severe dementia, using methods previously validated methods from Langa et al.[2] Specifically, we used criteria developed using data from the Asset and Health Dynamics (AHEAD) Study, a sub-study of the HRS. Langa et al. used the presence of an informal caregiver as a proxy for functional status and found that the cognitive assessment score thresholds they used tracked well with the

© 2021 Mafi JN et al. *JAMA Health Forum.*

presence of an informal caregiver. For example, patients with moderate dementia more frequently reported the presence of a caregiver than patients with mild dementia. Based on this work and in consultation with Dr. Kenneth Langa, the study's lead author, we also further classified dementia severity using the following threshold scores for the 27-point and 11-point scales, respectively (1) mild dementia: 5-6 and 6; (2) moderate dementia 3-4 and 7; and (3) severe dementia 0-2 and 8-11. Of note, because of some differences between the survey used in Langa's original paper and our 2016 HRS core survey used for our study, we used similar but not identical measures of dementia stage to Langa's measure. Working in consultation with Dr. Langa, we adapted our measures to match Langa's measures as closely as possible. Nonetheless, because these measures were similar but not identical to Langa's measures, we performed an additional sensitivity analysis to ensure that our own modified measures remained valid. Specifically, we used the presence of informal caregiver as a proxy for functional status limitations and thereby dementia stage, expecting that as dementia stage worsened, there would be a monotonic increase in the presence of an informal caregiver.[2,3] Using data from our 2016 HRS core sample on informal caregivers among non-institutionalized community-dwelling older adults, we found a strong relationship between our own measures of dementia stage and the presence of informal caregivers, shown in eTable 1.

Ultimately, when we quantified patients without using dementia staging, we did obtain similar results to the more refined estimates. For the upper bound, we identified 0.95 million eligible participants with MCI and 6.7 million eligible participants with MCI or any dementia. When we applied the more refined dementia staging estimates, we identified 1.1 million eligible participants with MCI and mild dementia and 5.7 million eligible participants with MCI and mild dementia. We chose the latter, more refined estimates to be more consistent with the revised FDA label, which stated that aducanumab should be prescribed in patients with AD-related MCI or mild dementia.

Of note, per NIA-AA guidelines we did not exclude vascular dementia and instead focused on amyloid plaque burden as a marker for treatment eligibility, given that AD often co-exists with other dementia types.[5,6]

© 2021 Mafi JN et al. *JAMA Health Forum.*

Cost Analysis Assumptions

Drug Costs

Using patient weight data from the 2016 HRS core sample, we used a patient weight-based drug cost method, more precise than the manufacturer's estimates, which had assumed average patient weight of 74 kg. While the HRS collects self-reported weights from nearly all participants, the HRS also provides actual weights for a subsample of respondents who participate in in-person interviews. To affirm the validity of the self-reported weights, we provide comparisons of summary statistics of self-reported versus actual weights among the subset of participants (n=7,733 participants) who provide both actual and self-reported weights. We also provide summary statistics for all participants who provide self-reported weights (n=20,684 participants). This exercise, shown below in eTable 2, shows similar distributions of patient weights between measured and self-reported weights. Summary statistics of self-reported versus actual patient weights were also similar among patients with MCI and dementia (data not shown). These sensitivity analyses suggest that self-reported patient weights are a reasonable proxy for actual patient weights, and these self-reported weights are what we incorporated to determine weight-based dosing.

Nevertheless, before we can explain how to incorporate these weights into our cost model, we must first explain the weight-based dosing costs for aducanumab, which have a nonlinear relationship with the patient's weight because the drug only comes in either 300 mg/3 ml or 170 mg/1.7 ml vials. In The manufacturer's press release in December 2021, aducanumab's $28,200 annual whole sale acquisition cost (e.g., "list price" [reduced from the previous $56,000 per year announced in June 2021]) was based on the assumption that the average patient with MCI or mild dementia would weigh 74 kilograms.[7,8] At full 10 mg/kg dosing per patient per administration, the most cost-efficient (e.g., the most inexpensive) dosing for a 74 kg patient would require two 300 mg/3 ml vials plus one 170 mg/1.7 ml vial, which yields 770 mg of drug per patient per monthly administration (740 mg used for the 74 kg patient and a small amount or 30 mg of drug wasted per month). To find the most cost-efficient vial combination, we created a loop algorithm in SAS version 9.4 to find the least expensive vial combination for each participant in the sample, based on each participant's self-reported weight. We then multiplied

© 2021 Mafi JN et al. *JAMA Health Forum.*

the cost of each unique vial combination for each participant in the sample by the survey-weighted proportion of beneficiaries in each patient weight category (see eTable 3).

Based on our real-world estimates of patient weights among Medicare Part B beneficiaries aged 65 years or older with MCI or mild dementia, the estimated annual drug acquisition cost will now increase from $28,200 to $27,759.36, assuming 12 monthly infusions per year. A detailed breakdown of the weight distribution and calculated corresponding drug acquisition costs of our upper-bound sample population using the most cost-efficient dosing strategy is included in eTable 3. Note that our dosing strategy minimizes costs, even if that resulted in the use of more vials per patient.

Associated Ancillary Health Services Costs

Per review of published FDA documents and reviews of clinical trial data, we assumed each patient would receive a one-time PET scan to screen for plaque, and, of those, 37%-68% would show substantial plaque burden based on population studies of patients with MCI and dementia.[5,6] That would leave between 32-63% of participants who would receive a negative PET scan, and therefore would not go on to receive aducanumab. To partly account for the cost of PET screening among those with negative scans, this would mean we would anticipate between 1/0.68=1.5 PET scans per patient and 1/0.37=2.7 PET scans per patient, respectively. We conservatively chose the lower bound estimate of 1.5 PET scans per patient-year. Of note, we assumed Medicare would decide to cover amyloid PET scans due to the FDA

© 2021 Mafi JN et al. *JAMA Health Forum.*

approval of aducanumab (Medicare does not currently cover this). Moreover, we anticipated few lumbar punctures due to patient risks and logistical barriers. Per FDA guidance, we also assumed 12 infusions of aducanumab per year (once every month), 1 routine geriatrics/neurology visit every 3 months, 2 routine monitoring MRIs per year (conservatively assuming baseline MRI is already done within past year) per patient-year.[9] Each infusion would also require a $198.12 infusion fee. This fee accounts for facility fees added to the professional fee approximately 50% of the time (see below for further details on facility fees). We estimated each patient with amyloid plaque on PET imaging would also receive Apo E serum testing once, since positive test results are associated with frequency of ARIA events and treatment response.[9]

We determined frequency of additional associated MRIs and specialist visits using the FDA's aggregated clinical trial data on ARIA frequency from two Phase 3 clinical trials. First, 26% of full dose treatment arm participants experienced moderate to severe ARIA, for which the FDA recommends a follow up MRI every 4 weeks until resolution of ARIA. Given the stakes of missing serious and potentially irreversible neurologic deficits, we assumed each of these MRIs would be accompanied by a neurologist or geriatrician evaluation at an office visit as well. We chose to use the rate of ARIA frequency at the full dose (10 mg/kg) because aducanumab patients are likely to be on the drug for longer than the duration of the clinical trial, meaning that the ramp-up period of dosage comparatively short overall. Because ARIA events last on average for 12 weeks, this would require approximately 3 additional MRIs and neurology/geriatrics visits every 4 weeks until moderate to severe ARIA resolved. This arithmetically translated to 0.26 x 3 = 0.78 or 0.78 additional MRIs and 0.78 additional neurology/geriatrics visits per patient-year due to moderate to severe ARIAs. Mild ARIAs also occurred in 15% of patients in the treatment arm. These require similar monitoring, but only when they are symptomatic. Because the FDA report did not directly present patient-level symptom rates among patients with mild ARIAs, we extrapolated symptom rates among all ARIAs, which are symptomatic 24% of the time overall. This translated to an estimated 0.15 x 0.24 = 3.6% of patient-years with mild ARIA and

© 2021 Mafi JN et al. *JAMA Health Forum.*

symptoms, and then 0.036 x 3 = 0.108 additional MRIs and visits per patient-year (using same assumptions of the number of follow up MRIs and visits per above), or in sum 0.108 additional MRIs and neurology/geriatrics visits per patient-year generated by mild symptomatic ARIAs. Serious adverse events associated with ARIAs occurred in 2% of treatment arm participants and would require hospitalization.

Costs were tabulated using the 2021 Medicare physician fee schedule and other publicly available sources (e.g., AHRQ Health Care Cost and Utilization Project[10]) using national average 2021 allowed amounts (including CPT codes 78608, 70551, and 99214 for PET brain, MRI brain, and neurology or geriatrics level 4 office visits respectively).[11] Further, using publicly available analyses from the Medicare Payment Advisory Commission on setting of care for U.S. chemotherapy infusions in 2019 (MedPAC's March 2021 Report to Congress[12]), we estimated that approximately 50% of infusion treatments would occur in hospital outpatient departments and approximately 50% would occur in private physician offices. Therefore, the final average unit cost for IV infusions applied non-facility professional fees 50% of the time and applied facility fees plus professional fees the remaining 50% of the time. We applied facility fees 15% of the time for all other remaining outpatient services such as neurology visits based on the MedPAC report finding that 15% of outpatient oncology visits occurred in hospital outpatient department settings.[12] In addition, to estimate facility fees for diagnostic imaging, we used published data from the Brookings Institution, which found that 69% of MRIs in 2018 occurred in the hospital outpatient department setting. Therefore, we incorporated facility fees to 70% of MRI and PET scan studies.[13] Finally, approximately 9% (9.1%) of trial participants dropped out of the treatment arm for any reason, including adverse drug events. To account for this general attrition, we subtracted 1% from the eligible population in each month during months 2-10 and multiplied by the estimated monthly cost to estimate the per patient annual cost to Medicare after patient attrition over time. Of note, the FDA label provides no time limit on the treatment duration. Also note that when we calculate the proportion of ancillary costs at the individual level, this reduces the amount to 19.1% of total costs compared with 19.4% of costs at the population level. This individual-level estimate of 19.1% is slightly lower than the population-level estimate of

© 2021 Mafi JN et al. *JAMA Health Forum.*

19.4% due to patient attrition. Specifically, this is because over 12 months' time, 1% of patients exit the population each month between months 2-10 (totaling 9% attrition per the clinical trials), and because months 2-10 have relatively higher drug costs compared with ancillary costs. Month 1 has the highest ancillary costs due to several baseline services such as the one-time baseline PET scan, which costs approximately $2,300 per patient per year ($1535 x 1.5, to partly account for the cost of PET screening among those with negative scans). In contrast, relatively fewer ancillary services occur during months 2-10 compared with month 1. As a verification test, when we removed patient attrition from our population cost model, the ancillary costs comprised 19.1% of total population cost estimates.

Out-of-pocket cost calculations

After reviewing CMS coverage rules, several published reports, and calling private payers, we confirmed out-of-pocket cost coverage rules for Medicare fee-for-service beneficiaries. Specifically, the Medicare beneficiaries in our sample without supplemental coverage are obligated to pay 20% coinsurance for Part B services, and so 20% of per-person annual costs we tabulated yielded $6,864.66.[14] Although this analysis includes both Medicare fee-for-service and Medicare Advantage beneficiaries, we estimated these out-of-pocket costs using traditional Medicare fee-for-service rules (80:20 ratio), but this coverage split may differ for Medicare Advantage patients. Per Medicare coverage policy rules, Medicare providers cannot bill dual-eligible Medicare-Medicaid beneficiaries who are Qualified Medicare Beneficiaries for Part A or B cost sharing, including deductibles, copays, and coinsurance.[15] This would effectively make cost-sharing for some dual-eligible beneficiaries $0, which forms the lower bound of our range of anticipated out-of-pocket costs. Nevertheless, we can say these beneficiaries will be expected to also pay out of pocket, but not less than $0 and not more than $6,864.66. The caveat, as noted above, is that Medicare Advantage beneficiaries might pay more (or less) than this upper limit, however, the total costs would remain the same regardless of the coverage split policy. Unfortunately, we cannot provide more precise estimates of out-of-pocket costs than an estimated range until private supplemental insurance and Medicare Advantage coverage policies are announced.

© 2021 Mafi JN et al. *JAMA Health Forum.*

eTable 1. Presence of Reported Informal Caregivers Stratified by Dementia Stage

Frequency Col Pct	*Normal*	*CIND*	*Mild Dementia*	*Moderate Dementia*	*Severe Dementia*	*Total*
**Frequency of Reported Informal Caregiver (%)**	1,455 (9.4)	805 (24.9)	166 (40.1)	97 (55.1)	220 (89.1)	2,743
**Total Sample**	15,544	3,234	414	176	247	19,615

© 2021 Mafi JN et al. *JAMA Health Forum.*

eTable 2. Comparison of Measured Weight and Self-Reported Weight Distributions Among HRS Participants in 2016

Variable	*N*	*Mean*	*Std Dev*	*Median*	*Min*	*Max*	*Lower Quartile*	*Upper Quartile*
**Measured Weight (kg)**	7,785	82.6	18.9	81.2	22.0	151.5	69.0	94.4
**Self-Reported Weight (kg)**	20,684	81.9	20.1	79.4	27.2	181.4	68.0	93.0
**Difference**	7,733	-1.40	4.52	-1.1	-54.8	61.0	-2.7	0.23

© 2021 Mafi JN et al. *JAMA Health Forum.*

eTable 3. Weight-Dependent Drug Dosage Calculated Costs

Self-Reported Weight (kg)	Number of 170 mg/1.7 mL vials	Number of 300 mg/3.0 mL vials	Percentage of Sample (%)	Calculated Cost for Full Dose ($)
< 47	1	1	2.6073	1,325.40
47 – 51	3	0	3.1701	1,438.20
51 – 60	0	2	12.7211	1,692.00
60 – 64	2	1	7.9419	1,804.80
64 – 68	4	0	6.0514	1,917.60
68 – 77	1	2	20.1887	2,171.40
77 – 81	3	1	9.2681	2,284.20
81 – 85	5	0	8.7067	2,397.00
85 – 90	0	3	7.9584	2,538.00
90 – 94	2	2	5.4398	2,650.80
94 – 98	4	1	3.7038	2,763.60
98 – 102	6	0	3.5449	2,876.40
102 – 107	1	3	3.0897	3,017.40
107 – 111	3	2	2.1799	3,130.20
111 – 115	5	1	1.1082	3,243.00
115 – 119	7	0	0.5491	3,355.80
119 – 120	0	4	0.0246	3,384.00
120 – 124	2	3	0.4148	3,496.80
124 – 128	4	2	0.6835	3,609.60
128 – 132	6	1	0.1152	3,722.40
132 – 136	8	0	0.0436	3,835.20
136 – 137	1	4	0.1384	3,863.40
137 – 141	3	3	0.0000	3,976.20
141 – 145	5	2	0.0618	4,089.00
145 – 149	7	1	0.2152	4,201.80
149 – 150	0	5	0.0529	4,230.00
> 150	9	0	0.0207	4,314.60

© 2021 Mafi JN et al. *JAMA Health Forum.*

eReferences

1. Crimmins EM, Kim JK, Langa KM, Weir DR. Assessment of cognition using surveys and neuropsychological assessment: the Health and Retirement Study and the Aging, Demographics, and Memory Study. *J Gerontol B Psychol Sci Soc Sci.* 2011;66 Suppl 1:i162-171.
2. Langa KM, Chernew ME, Kabeto MU, et al. National estimates of the quantity and cost of informal caregiving for the elderly with dementia. *J Gen Intern Med.* 2001;16(11):770-778.
3. Hux MJ, O'Brien BJ, Iskedjian M, Goeree R, Gagnon M, Gauthier S. Relation between severity of Alzheimer's disease and costs of caring. *CMAJ.* 1998;159(5):457-465.
4. Understanding Medicare Advantage & Medicare Drug Plan Enrollment Periods. Centers for Medicare and Medicaid Services (CMS). https://www.medicare.gov/Pubs/pdf/11219-understanding-medicare-part-c-d.pdf. Accessed 12-14-2021.
5. Doraiswamy PM, Sperling RA, Johnson K, et al. Florbetapir F 18 amyloid PET and 36-month cognitive decline: a prospective multicenter study. *Mol Psychiatry.* 2014;19(9):1044-1051.
6. Petersen RC, Aisen P, Boeve BF, et al. Mild cognitive impairment due to Alzheimer disease in the community. *Ann Neurol.* 2013;74(2):199-208.
7. Biogen and Eisai launch multiple initiatives to help patients with Alzheimer's disease access ADUHELM™. Biogen. . https://investors.biogen.com/news-releases/news-release-details/biogen-and-eisai-launch-multiple-initiatives-help-patients. Accessed 10-23-2021.
8. Biogen Announces Reduced Price for ADUHELM® to Improve Access for Patients with Early Alzheimer's Disease. Biogen. Investor Relations. Published December 2021. https://investors.biogen.com/news-releases/news-release-details/biogen-announces-reduced-price-aduhelmr-improve-access-patients. Accessed 12-20-2021.
9. Center for Drug Evaluation and Research Clinical Review of Aducanumab. Food and Drug Administration (FDA). https://www.biogencdn.com/us/aduhelm-pi.pdf. Published 2021. Accessed 08-10-2021.
10. Medicare Advantage Versus the Traditional Medicare Program: Costs of Inpatient Stays, 2009–2017. Agency for Healthcare Research and Quality (AHRQ) Health Care Cost and Utilization Project (HCUP). https://hcup-us.ahrq.gov/reports/statbriefs/sb262-Medicare-Advantage-Costs-2009-2017.pdf. Published 2020. Accessed 09-25-2021.
11. Medicare Physician Fee Schedule Look Up Tool. Centers for Medicare and Medicaid Services (CMS). https://www.cms.gov/apps/physician-fee-schedule/search/search-criteria.aspx. Accessed 09-27-2021.
12. Report to the Congress: Medicare Payment Policy. Medicare Payment Advisory Commission (MedPAC). 2021.
13. Steinwald B, Ginsburg PB, Brandt C, Lee S. Medicare Advanced Imaging Payment: Dysfunctional Policy Making. USC-Brookings Schaeffer Initiative for Health Policy. https://www.brookings.edu/wp-content/uploads/2021/03/Imaging_Paper_Final.pdf. Published 2021. Accessed 10-23-2021.
14. Centers for Medicare and Medicaid Services (CMS) Medicare Costs at a Glance. https://www.medicare.gov/your-medicare-costs/medicare-costs-at-a-glance. Accessed 09-25-2021.
15. Centers for Medicare and Medicaid Services (CMS) Dually Eligible Individuals - Categories. https://www.cms.gov/Medicare-Medicaid-Coordination/Medicare-and-Medicaid-Coordination/Medicare-Medicaid-Coordination-Office/Downloads/MedicareMedicaidEnrolleeCategories.pdf. Accessed 09-25-2021.

© 2021 Mafi JN et al. *JAMA Health Forum.*
